# [^18^F]AV‐1451 binding is increased in frontotemporal dementia due to C9orf72 expansion

**DOI:** 10.1002/acn3.631

**Published:** 2018-09-14

**Authors:** Richard W. Bevan‐Jones, Thomas E. Cope, Simon P. Jones, Luca Passamonti, Young T. Hong, Tim Fryer, Robert Arnold, Jonathan P. Coles, Franklin A. Aigbirhio, Karalyn Patterson, John T. O'Brien, James B. Rowe

**Affiliations:** ^1^ Department of Psychiatry University of Cambridge Cambridge United Kingdom; ^2^ Department of Clinical Neurosciences University of Cambridge Cambridge United Kingdom; ^3^ Wolfson Brain Imaging Centre University of Cambridge Cambridge United Kingdom; ^4^ Division of Anaesthesia University of Cambridge Cambridge United Kingdom; ^5^ Medical Research Council Cognition and Brain Sciences Unit Cambridge United Kingdom

## Abstract

The PET ligand [^18^F]AV‐1451 was developed to bind tau pathology in Alzheimer's disease, but increased binding has been shown in both genetic tauopathies and in semantic dementia, a disease strongly associated with TDP‐43 pathology. Here we assessed [^18^F]AV‐1451 binding in behavioral variant frontotemporal dementia due to a hexanucleotide repeat expansion in C9orf72, characterized by TDP‐43 pathology. We show that the C9orf72 mutation increases binding in frontotemporal cortex, with a distinctive distribution of binding compared with healthy controls.

## Introduction

[^18^F]AV‐1451 was developed as a specific marker of paired helical filament tau (PHF‐tau) pathology in Alzheimer's disease (AD), and is selective for PHF‐tau over beta‐amyloid and alpha‐synuclein in vitro.^1^ In vivo studies have confirmed elevated and distributed binding in keeping with typical and atypical presentations of Alzheimer's disease,^2^, ^3^ consistent with Braak staging.^4^ Binding characteristics in neurodegenerative diseases other than Alzheimer's disease are controversial.

In vivo and *post mortem* studies indicate increased [^18^F]AV‐1451 binding in patients with frontotemporal dementia due to mutations in the microtubule‐associated protein tau (MAPT), albeit with differences between mutations associated with paired helical filament tauopathy and straight filament tauopathy.^5^, ^6^ Regionally elevated binding also occurs in another tauopathy, progressive supranuclear palsy.^7^, ^8^ However, [^18^F]AV‐1451 binding is also seen in patients with semantic dementia, a disease strongly associated with TAR DNA‐binding Protein‐43 (TDP‐43), especially type C.^9^, ^10^ The non‐tau target of [^18^F]AV‐1451 binding in semantic dementia is unknown and, whilst *post mortem* studies suggest a lack of binding to TDP‐43 pathology,^11^, ^12^, ^13^ the elevated binding seen in vivo is in keeping with the characteristic anatomical distribution of neuropathology in semantic dementia.

A limitation of previous studies of [^18^F]AV‐1451 binding in semantic dementia cases is their reliance on clinico‐pathological correlations to interpret the neuroimaging data. In this report, we describe [^18^F]AV‐1451 binding in a patient with a clear familial case of frontotemporal dementia (FTD) due to a hexanucleotide repeat expansion in the C9orf72 gene. This is the commonest genetic cause of FTD^14^ and it is strongly associated with TDP‐43 pathology^15^ without the presence of any tau. We examine not only the magnitude of the [^18^F]AV‐1451 binding, but also the pattern of regional distribution across cortical and subcortical structures.

## Case and Controls

A 53‐year‐old man presented with a 3‐year history of progressive behavioral change characterized by disinhibition, grandiosity, and stereotyped behaviors. He subsequently developed semantic impairments and anomia, with loss of single word comprehension and surface dyslexia. At the time of imaging, he scored 53/100 on the Addenbrooke's Cognitive Examination – revised edition (ACE‐R, reference range >88) and 25/30 on the Mini Mental State Examination (MMSE). There were significant carer endorsements for changes in memory, challenging behaviors, altered eating habits and abnormal beliefs on the revised Cambridge Behavioural Inventory. Structural magnetic resonance imaging of his brain confirmed frontotemporal atrophy. His maternal uncle had died of motor neurone disease. Genetic testing confirmed a hexanucleotide repeat expansion in C9orf72. He met diagnostic criteria for definite behavioral variant frontotemporal dementia.^16^, ^17^ He underwent research structural MRI and [^18^F]AV‐1451 positron emission tomography (PET) as part of the Neuroimaging of Inflammation in Memory and Related Other Disorders (NIMROD) study.^18^ Thirteen healthy volunteers acted as controls (age range 55–80, 6 Male, ACE‐R range 89–99, MMSE range 28–30) and underwent the same neuroimaging and behavioral protocol.

## Data Modeling and Statistical Method

When comparing the case with the C9orf72 genetic expansion and controls, two questions were posed. First, were there regions of the brain with increased nondisplaceable‐binding potential (BP_ND_)? Secondly, irrespective of the absolute level of ligand binding, did the distribution of binding across brain regions differ? This second question focusses on the multivariate distribution or pattern of binding, in relation to the distribution of neuropathological substrates of frontotemporal dementias. The pattern may be abnormal, even where no single region on its own has particularly high binding of [^18^F]AV‐1451.

To address the first question, the brain was parcellated into 83 regions, using the Hammersmith atlas n30r83 modified to include some additional subcortical structures.^7^ The [^18^F]AV‐1451 BP_ND_ in each region was calculated using the methods described in Passamonti et al.^7^ to obtain regional BP_ND_ which were adjusted for cerebrospinal fluid (CSF) partial volume effects. Individual *t*‐tests were then performed in each region to compare the observed [^18^F]AV‐1451 BP_ND_ in the C9orf72 case and controls, correcting for multiple comparisons across the full data range (FDR correction, *P* < 0.05, plus illustration at the more liberal threshold of *P* < 0.05 uncorrected).

To address the second question, a hierarchical cluster analysis was used. The CSF corrected and parcellated [^18^F]AV‐1451 BP_ND_ data were then converted to individual linear vectors containing all regions of interest. The pairwise Spearman's rank order correlations were calculated between all subjects, and the inverse taken to produce a dissimilarity matrix. This represents the difference in [^18^F]AV‐1451 BP_ND_ distribution between every pair of subjects, blinded to the absolute level of that binding. These dissimilarity measures were used to calculate a linkage dendrogram using an average distance method.

We also compared regional gray matter volume between the case and control group. Regional gray matter volumes were calculated during PET processing and parcellated, using the same atlas. For each region, linear models were used with region volume, age and total intracranial volume as covariates.^19^ *T*‐tests were performed between the residual of the patient's volume and predicted volume, based on the controls’ regression, and the control residuals. The *P*‐values for each test were increased to compensate for the degrees of freedom used in the control regression. False discovery rate (FDR) correction was then applied to these adjusted *P*‐values in the same manner as the [^18^F]AV‐1451 BP_ND._

## Results

### [^18^F]AV‐1451 in C9orf72 FTD versus controls

**Figure 1:**
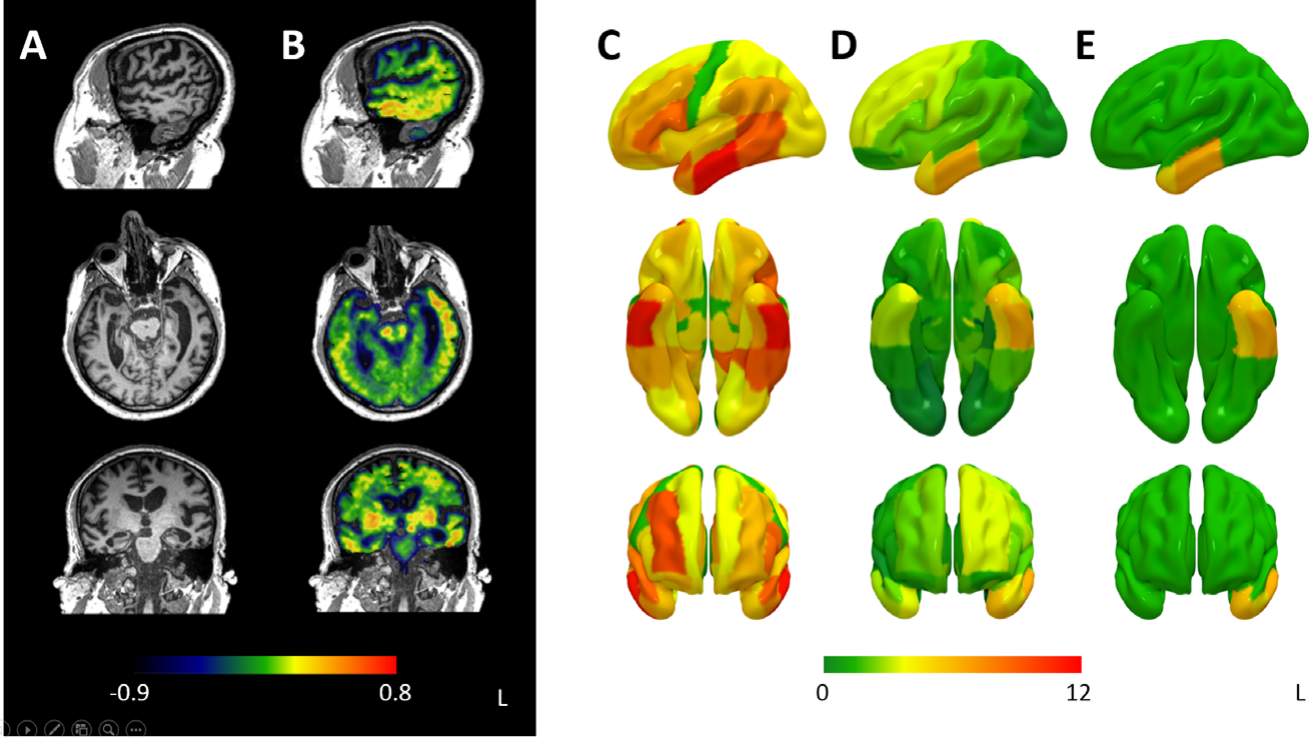
Panel A shows selected sagittal, coronal and axial slices from the structural MRI (T1‐weighted MPRAGE) of the C9orf72 case. Panel B shows the [^18^F]AV‐1451‐binding potential (BP_ND_) without correction for partial volume effects in the same planes. Panel C shows the unthresholded t‐scores for gray matter atrophy on a volumetric rendering on the smoothed MNI152 template MRI. Panel D shows unthresholded t‐scores for (BP_ND_) in the same way. Panel E shows the same data but thresholded at p < 0.05 corrected for false discovery rate.

T1‐weighted MPRAGE images are shown in Figure 1A and raw BP_ND_ images uncorrected for partial volume effects in Figure 1B. The left frontotemporal regions demonstrated increased BP_ND_ in the C9orf72 case compared to controls. After correcting for multiple comparisons (*P* < 0.05 FDR), significant abnormalities were observed in left fusiform gyrus, left medial anterior temporal lobe, left middle and inferior temporal gyri, and left lateral inferior temporal lobe (Fig. 1E). In addition to this, more liberal thresholding without correction for multiple comparisons demonstrated bilateral changes, with elevated BP_ND_ also found in the right medial anterior temporal lobe, left anterior superior temporal gyrus, superior and middle frontal gyri, pallidum, and substantia nigra (Fig. 1D).

The distribution of BP_ND_ was also clearly dissimilar in the C9orf72 case compared to all controls (Figure S1A). The hierarchical clustering analysis indeed classified the patient as an outlier compared to controls (Figure S1B).

The regional volume analysis revealed widespread gray matter loss, most marked in the temporal lobes, which remained after FDR correction (Fig. 1C). *T*‐scores along with FDR corrected *P*‐values for both [^18^F]AV‐1451 and gray matter volume are shown in Table S1.

## Discussion

**Figure 2:**
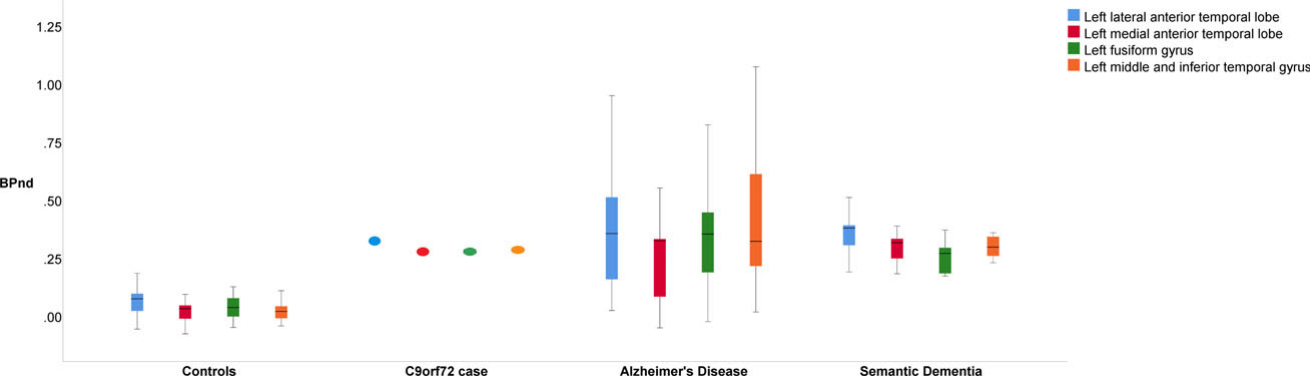
Boxplots of the nondisplaceable [^18^F]AV‐1451‐binding potential in those regions of interest that are significantly elevated in the C9orf72 case compared to controls after FDR correction. As well as data for the C9orf72 case and the control population, comparative data are shown using the same analysis methods for our previously published cohorts with Alzheimer's disease^7^ and Semantic Dementia^9^.

C9orf72 expansions are strongly associated with TDP‐43 type B and dipeptide repeat pathology. We therefore infer that at 53 years old, with the C9orf72 expansion, and a classical FTD presentation, the present case has TDP‐43 pathology without tauopathy. Despite this, the participant exhibited elevated [^18^F]AV‐1451 binding in frontotemporal regions of a magnitude greater than the 95th centile of binding in controls but within the interquartile range for binding in Alzheimer's disease^7^ and semantic dementia^9^ (Fig. 2). Combined with similar findings in 14 cases with semantic dementia,^9^, ^10^ this suggests that the [^18^F]AV‐1451 PET ligand is not specific for tau over TDP‐43 pathology in Frontotemporal lobar degeneration. Therefore, while [^18^F]AV‐1451 might retain a role in tracking disease by visualising the distribution and/or severity of neuropathology in a person with frontotemporal dementia, it is unlikely to have utility for cohort selection for disease modifying trials that target tau or TDP‐43‐specific disease mechanisms.

As expected in this participant with moderate dementia from a C9orf72 expansion, there was widespread gray matter loss with a predilection for frontotemporal regions, and particularly temporal regions in keeping with his clinical syndrome. The regions of elevated BP_ND_ with largest t‐scores were situated within the atrophic regions but elevated BP_ND_ did not occur across all atrophic regions. The colocation of elevated BP_ND_ and atrophy is perhaps unsurprising if [^18^F]AV‐1451 is binding to a nontau molecule associated with C9orf72‐related neurodegeneration.

There are limitations in this study. First we have analysed data from a single case against a control group. Larger studies in C9orf72 FTD might expand upon these results, as C9orf72 frontotemporal dementia is clinically and neuroanatomically heterogeneous.^20^ While our C9orf72 case is young, mutation positive, and meets diagnostic criteria for definite behavioral variant frontotemporal dementia, we lack biomarker or pathology proof of the absence of coincidental Alzheimer tau pathology that may occur in a small percentage of adults in their fifties. However, age‐related presymptomatic Alzheimer pathology is rendered an unlikely explanation for our data, both by the low population prevalence at 53, and the regions found to have elevated binding in our case which are clearly distinct from those usually observed in Alzheimer's disease. Finally, we note that the calculation of [^18^F]AV‐1451 BP_ND_ data uses the superior cerebellar gray matter as a reference region, as in our other recent papers.^5^, ^7^, ^9^ Although across most of the genetic and sporadic forms of FTD this region remains unaffected, in previous cases with C9orf72 expansion, cerebellar atrophy and dipeptide aggregation have been described.^21^ In mitigation of this potential shortcoming, whilst as expected there was cerebellar gray matter atrophy in the present C9orf72, there was no observable elevated cerebellar [^18^F]AV‐1451 signal in the uncorrected PET BP_ND_ map. Moreover, if elevated binding in this reference cerebellar region were present, it would have only reduced the estimated BP_ND_ elsewhere, making the risk of false negative results more likely. In any case, it is important to note that the level of cerebellar binding does not affect the non‐parametric analysis of distributional‐binding differences.

Elevated binding in genetic TDP‐43‐associated FTD provides further evidence for anatomically specific binding of [^18^F]AV‐1451 to non‐tau targets. However, the sensitivity to longitudinal changes in TDP‐43‐associated FTD, and the molecular identity of the [^18^F]AV‐1451 target, remain to be determined.

## Author Contributions

WRBJ wrote the first draft, all other authors provided review and critique of manuscript. JBR, JTOB, FIA, TDF and KP conceptualized and designed the study. WRBJ, TDF, YTH and JPC were integral to the organization and execution of the study. WRBJ, TEC and PSJ designed and executed the statistical analysis, KP, LP and JBR provided statistical review and critique.

## Conflict of Interest

JOB has acted as a consultant for GE Healthcare and Lilly.

## Supporting information


**Figure S1.** (A) Spearman dissimilarity matrix (1‐correlation) between all individuals. The first row and column, separated by black lines from the other rows and columns, represents the patient. The other thirteen columns represent controls. (B) the average linkage dendrogram produced by hierarchical cluster analysis. The two resultant clusters are colored in red and black.

**Table S1**. Displaying T‐scores and FDR corrected p‐values for [^18^F]AV‐1451‐binding potential and for atrophy in each region, ordered by magnitude of [^18^F]AV‐1451‐binding potential T‐score.
