# Supplementary figures and images for: [18F]AV‐1451 binding is increased in frontotemporal dementia due to C9orf72 expansion
Source: Ann Clin Transl Neurol. 2018 Sep 14;5(10):1292–6. doi: 10.1002/acn3.631 (PMC6186940; doi:10.1002/acn3.631)

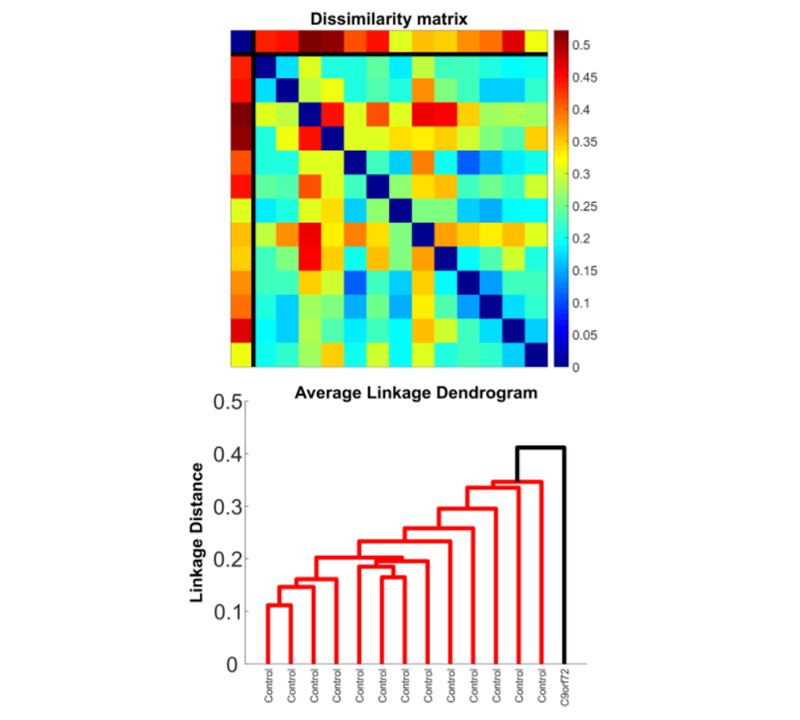

Supplement: Supplementary file 1 — Figure S1. (A) Spearman dissimilarity matrix (1‐correlation) between all individuals. The first row and column, separated by black lines from the other rows and columns, represents the patient. The other thirteen columns represent controls. (B) the average linkage dendrogram produced by hierarchical cluster analysis. The two resultant clusters are colored in red and black. [file ACN3-5-1292-s001.png]
