# Supplementary material for: [18F]AV‐1451 binding is increased in frontotemporal dementia due to C9orf72 expansion
Source: Ann Clin Transl Neurol. 2018 Sep 14;5(10):1292–6. doi: 10.1002/acn3.631 (PMC6186940; doi:10.1002/acn3.631)
Supplement: Supplementary file 2 — Table S1. Displaying T‐scores and FDR corrected p‐values for [18F]AV‐1451‐binding potential and for atrophy in each region, ordered by magnitude of [18F]AV‐1451‐binding potential T‐score. [file ACN3-5-1292-s002.docx]

| **Region name** | **AV T score** | **AV FDR corrected p value** | **Atrophy T score** | **Volume FDR corrected p value** |
| --- | --- | --- | --- | --- |
| Left middle and inferior temporal gyrus | 5.98 | 0.01 | 11.40 | 0.000 |
| Left anterior temporal lobe medial part | 5.19 | 0.01 | 4.59 | 0.002 |
| Left fusiform gyrus | 4.20 | 0.03 | 6.24 | 0.001 |
| Left anterior temporal lobe lateral part | 3.95 | 0.04 | 6.75 | 0.000 |
| Left superior temporal gyrus anterior part | 3.60 | 0.06 | 8.61 | 0.000 |
| Left pallidum | 2.83 | 0.17 | 0.02 | 0.987 |
| Left substantia nigra | 2.78 | 0.17 | 2.07 | 0.078 |
| Right anterior temporal lobe medial part | 2.58 | 0.22 | 5.25 | 0.001 |
| Left superior frontal gyrus | 2.44 | 0.25 | 2.75 | 0.029 |
| Left middle frontal gyrus | 2.40 | 0.25 | 5.62 | 0.001 |
| Left precentral gyrus | 1.99 | 0.42 | 2.26 | 0.059 |
| Right anterior temporal lobe lateral part | 1.83 | 0.46 | 6.18 | 0.001 |
| Right middle and inferior temporal gyrus | 1.70 | 0.51 | 11.27 | 0.000 |
| Right fusiform gyrus | 1.68 | 0.51 | 4.61 | 0.002 |
| Right presubgenual frontal cortex | 1.62 | 0.51 | 4.10 | 0.004 |
| Left cerebellum dentate | 1.54 | 0.52 | 3.08 | 0.018 |
| Left putamen | 1.35 | 0.64 | 2.24 | 0.059 |
| Left gyrus cinguli posterior part | 1.26 | 0.67 | 3.37 | 0.011 |
| Right superior frontal gyrus | 1.23 | 0.67 | 2.55 | 0.038 |
| Left inferior frontal gyrus | 1.11 | 0.74 | 8.30 | 0.000 |
| Right superior temporal gyrus anterior part | 1.08 | 0.74 | 8.62 | 0.000 |
| Right substantia nigra | 1.05 | 0.74 | 1.86 | 0.109 |
| Left insula | 1.05 | 0.74 | 4.51 | 0.003 |
| Left amygdala | 1.04 | 0.74 | 4.96 | 0.001 |
| Left subgenual frontal cortex | 0.93 | 0.77 | 2.66 | 0.033 |
| Right nucleus accumbens | 0.91 | 0.77 | 0.16 | 0.887 |
| Left posterior orbital gyrus | 0.90 | 0.77 | 5.61 | 0.001 |
| Right middle frontal gyrus | 0.87 | 0.77 | 9.21 | 0.000 |
| Right putamen | 0.85 | 0.77 | 1.11 | 0.325 |
| Left inferiolateral remainder of parietal lobe | 0.82 | 0.77 | 6.23 | 0.001 |
| Left thalamus | 0.80 | 0.77 | 4.07 | 0.004 |
| Left superior temporal gyrus posterior part | 0.77 | 0.79 | 5.06 | 0.001 |
| Left posterior temporal lobe | 0.74 | 0.80 | 8.69 | 0.000 |
| Left postcentral gyrus | 0.70 | 0.81 | 3.06 | 0.018 |
| Right medial orbital gyrus | 0.66 | 0.83 | 3.55 | 0.009 |
| Right subgenual frontal cortex | 0.64 | 0.83 | 2.26 | 0.059 |
| Left medial orbital gyrus | 0.59 | 0.87 | 3.49 | 0.010 |
| Left nucleus accumbens | 0.52 | 0.89 | 0.36 | 0.745 |
| Right pallidum | 0.36 | 0.95 | 1.27 | 0.259 |
| Left caudate nucleus | 0.33 | 0.95 | 2.56 | 0.038 |
| Right straight gyrus | 0.33 | 0.95 | 2.83 | 0.026 |
| Right thalamus | 0.32 | 0.95 | 3.60 | 0.008 |
| Right caudate nucleus | 0.32 | 0.95 | 0.65 | 0.567 |
| Right anterior orbital gyrus | 0.27 | 0.95 | 5.29 | 0.001 |
| Right amygdala | 0.24 | 0.95 | 5.51 | 0.001 |
| Left straight gyrus | 0.20 | 0.96 | 4.53 | 0.003 |
| Left lateral orbital gyrus | 0.16 | 0.97 | 5.20 | 0.001 |
| Bilateral brainstem pons | 0.11 | 0.99 | 5.19 | 0.001 |
| Right cerebellum grey matter | 0.08 | 0.99 | 4.83 | 0.002 |
| Right gyrus cinguli posterior part | 0.07 | 0.99 | 3.42 | 0.011 |
| Left cingulate gyrus anterior part | 0.06 | 0.99 | 6.12 | 0.001 |
| Right lateral orbital gyrus | 0.05 | 0.99 | 3.92 | 0.005 |
| Left superior parietal gyrus | -0.02 | 0.99 | 2.64 | 0.034 |
| Left cerebellum grey matter | -0.02 | 0.99 | 5.29 | 0.001 |
| Right subcallosal area | -0.03 | 0.99 | 0.71 | 0.540 |
| Left presubgenual frontal cortex | -0.17 | 0.97 | 2.43 | 0.045 |
| Right precentral gyrus | -0.25 | 0.95 | 1.28 | 0.259 |
| Bilateral brainstem midbrain | -0.25 | 0.95 | 3.76 | 0.007 |
| Left anterior orbital gyrus | -0.28 | 0.95 | 5.09 | 0.001 |
| Right parahippocampal and ambient gyri | -0.37 | 0.95 | 2.83 | 0.026 |
| Right superior parietal gyrus | -0.38 | 0.95 | 4.06 | 0.004 |
| Right insula | -0.39 | 0.95 | 5.63 | 0.001 |
| Right inferior frontal gyrus | -0.48 | 0.92 | 4.00 | 0.005 |
| Right posterior orbital gyrus | -0.52 | 0.89 | 4.06 | 0.004 |
| Right cingulate gyrus anterior part | -0.52 | 0.89 | 5.38 | 0.001 |
| Right posterior temporal lobe | -0.81 | 0.77 | 5.82 | 0.001 |
| Left lateral remainder of occipital lobe | -0.87 | 0.77 | 3.84 | 0.006 |
| Left subcallosal area | -0.89 | 0.77 | 5.42 | 0.001 |
| Right postcentral gyrus | -0.91 | 0.77 | 5.94 | 0.001 |
| Right inferiolateral remainder of parietal lobe | -1.15 | 0.73 | 5.61 | 0.001 |
| Bilateral brainstem medulla | -1.23 | 0.67 | 4.17 | 0.004 |
| Left parahippocampal and ambient gyri | -1.26 | 0.67 | 3.05 | 0.018 |
| Right superior temporal gyrus posterior part | -1.44 | 0.58 | 7.81 | 0.000 |
| Left lingual gyrus | -1.57 | 0.52 | 2.53 | 0.039 |
| Right lateral remainder of occipital lobe | -1.57 | 0.52 | 2.79 | 0.027 |
| Right cerebellum dentate | -1.63 | 0.51 | 1.97 | 0.092 |
| Right hippocampus | -1.82 | 0.46 | 1.38 | 0.229 |
| Right cuneus | -1.95 | 0.42 | 0.69 | 0.546 |
| Right lingual gyrus | -1.96 | 0.42 | 5.07 | 0.001 |
| Left cuneus | -2.16 | 0.36 | 0.63 | 0.571 |
| Left hippocampus | -3.16 | 0.11 | 5.66 | 0.001 |

**Supplementary table 1**: Displaying T-scores and FDR corrected p-values for [^18^F]AV-1451 binding potential and for atrophy in each region, ordered by magnitude of [^18^F]AV-1451 binding potential T-score.
